# Bridging the gap: enhancing patient and public involvement in dermatology PRO research

**DOI:** 10.1186/s41687-026-01069-2

**Published:** 2026-05-29

**Authors:** Ella Brookes, Efstathios Zikos, Laurence Lucats, Loic Notelet, Giorgio Ferrari, Arnaud Magnier, Benoit Arnould

**Affiliations:** 1 Sanofi, Reading, UK; 2 Sanofi, Gentilly, France; 3 Sanofi, Milan, Italy

## Introduction

The landscape of clinical research is evolving rapidly, with patient-reported outcomes (PROs) increasingly recognized as essential components of treatment evaluation. In Dermatology, where visible symptoms and quality of life (QoL) impacts are profound, PROs offer critical insights beyond clinician assessments. However, a significant gap exists between regulatory requirements, patient priorities, and implementation practices. This editorial discusses the current state of Patient and Public Involvement and Engagement (PPIE) in dermatology PRO research, highlighting current challenges and proposing innovative approaches to enhance meaningful patient participation [1].

## The current landscape

Dermatology clinical trials face unique challenges in PRO implementation, dermatological conditions present as visible signs and symptoms which historically have been assessed by clinicians. The increasing emphasis on the ‘subjective suffering’ using patient-reported concepts based on patients’ perception of symptoms and reducing reliance on clinician assessment of observable signs brings new challenges. While validated tools exist for symptoms (primarily single-item Numerical Rating Scales), impacts on QoL are largely assessed using generic legacy instruments and their recognition varies among the recipients: health authorities, Health Technology Assessment (HTA) bodies, payers, healthcare professionals and patients. Upon examination of recent market authorizations for dermatology products, a disconnect has been revealed: QoL impacts are considered highly important to patients but not equally valued by regulators, payers, and HTA bodies [2, 3].

This disconnect creates a paradoxical situation where PROs capturing concepts most meaningful to those living with dermatological conditions receive less emphasis in regulatory and healthcare decision-making. The fast pace of conducting early clinical development may impede collaboration with patient communities until later phases of development, suggesting that PPIE often occurs too late in the research process to meaningfully influence study design and endpoint selection [4].

## Challenges in implementing meaningful PPIE

Several barriers impede effective PPIE in dermatology PRO research. First, the “must-have” COAs vary among recipient health authorities, HTA bodies and payers. The resulting long list of “must-have” COAs leaves little room for innovation, creating pressure on the COA strategy to perform and limits opportunities to demonstrate the value of innovation. Given the important consideration to reduce patient burden with respect to PRO completion, these existing requirements limit opportunities to incorporate additional or novel patient-centered PROs which might better capture day-to-day experiences of patients affected by dermatological conditions.

Second, the heterogeneity, both of patient populations as well as the dermatological conditions themselves, present important methodological challenges. Dermatology patients experience a wide range of impacts on QoL and various comorbidities, making standardized assessment less relevant. This patient heterogeneity demands more nuanced approaches to PRO development which can only be achieved through deeper and earlier patient engagement.

Third, operational constraints often limit PPIE implementation. The majority of dermatology clinical trials include adult patients first, then adolescent patients once sufficient safety is demonstrated followed by pediatric patients. This staggered approach in age enrollment in drug development results in underrepresentation of pediatric patients, adolescents, and their parents/guardians in conceptual modelling, PRO development and validation which is initiated at the beginning of a drug development.

## Innovative approaches to enhance PPIE

Despite these challenges, several approaches can enhance PPIE in dermatology PRO research.

### Early and continuous engagement with patients

A strong recommendation is to engage as early as possible with patient communities to build trust and encourage meaningful collaboration in the development of dermatology trials. This approach should be systematic, through formal frameworks that integrate patient perspectives from concept development through implementation and interpretation. Given the heterogeneity of patients and dermatological conditions, engagement strategies tailored to each condition not only acknowledge the unique challenges and priorities of diverse patient populations, but also aim to identify and understand the concepts that matter most to each patient group [5]. Early engagement allows patient priorities not only to inform specific clinical research questions and endpoint selection in protocols, but also to shape the entire clinical development strategy [3].

### Technology-enhanced participation

Early validation of PROs to support patient-centered endpoints, with diverse patient input including adolescents, pediatric and parents/guardians, can help support their acceptability while ensuring they capture patient meaningful outcomes. Clinical research should consider validating relevant PROs in a given dermatology indication early in clinical development at risk.

The use of technology in the design of the e-diary makes it more fun (for pediatric and adolescent patients) and may improve adherence in the completion of PROs. Digital solutions can reduce patient burden while increasing engagement, particularly for younger populations. Patient co-design of electronic PRO interfaces could improve completion rates and data quality while ensuring PROs capture relevant concepts meaningful to patients [6].

### Cross-functional collaboration

There is a need to work closely with internal organizational stakeholders to ensure continuity of partnerships with patient communities beyond early clinical development. Breaking silos between organizational teams which may include; clinical development, patient engagement, health economics, market access and public affairs teams can create more comprehensive PPIE strategies that span the product lifecycle.

To meaningfully engage and advance PPIE in dermatology PRO research, several systemic changes are needed.

First, regulatory agencies should provide additional guidance on acceptable methodologies for incorporating PPIE into clinical development and validation with the view of incorporating patient centric information in labelling leading the path to consistent requirements between PRO recipients [7]. The current lack of standardization in regulatory requirements and reliance on generic PROs creates uncertainty that discourages innovation.

Second, researchers and pharmaceutical companies should establish formal frameworks for evaluating the impact of PPIE on research quality and outcomes. Documenting feedback from regulators and ethics committees specific to PROs, for each dermatology indication, should extend to documenting the influence of patient input and relevance of concepts captured and how this should be considered during study design and interpretation.

The lack of patient involvement in dermatology clinical research has profound consequences not only for patients, but for pharmaceutical companies and clinicians alike. Patients can play a critical role in the following;

- Study design issues: without patient input there is a possibility to overlook crucial aspects of disease experience impacting applicability to real-world and uptake of treatment.
- Recruitment, retention and data quality issues: burdensome procedures and logistical challenges increase likelihood of dropouts, missing data and reduced adherence to procedures resulting in operational pressure, extended study timelines and compromised study data.
- Ethical considerations: active engagement and meaningful involvement of the patient community helps to build trust, lack of trust can impact future recruitment efforts and stakeholder relationships.

Recommendations from a recently published commentary [8] include working with local communities and organisations to encourage involvement of patients and other stakeholders in trial methodology. It also recommends monitoring best practices and engagement strategies, and providing regular feedback to trial sites, local community groups and patients to highlight the benefit of their participation and provide valuable feedback. Demonstrating meaningful patient engagement in practice, a global dermatology patient organization alliance implemented a Delphi panel approach including over 1000 globally recruited patients to inform the selection of the most relevant items and refine the key concepts of interest in support of the development of a novel dermatology-specific impact PRO [10]. In other therapeutic areas there are positive examples of the role patients played in identifying previously overlooked disease manifestations and treatment priorities. Barger et al. [9] describes how patient input influenced the study design of an oncology study, improved patient resource materials and identified important treatment relevant questions previously overlooked.

Finally, greater collaboration between stakeholders—including patients and patient organizations (i.e. Patient Advocacy Groups), academic institutions, industry, regulators, ethics committees is needed to align on the value of PPIE in informing the development of dermatology trials and inclusion of COAs. The current disconnect between patient priorities and regulatory requirements undermines the potential for PROs to be recognized as a major aspect of patient-centered care.

## Conclusion

Enhancing PPIE in dermatology PRO research requires moving beyond tokenistic involvement of patients toward a more robust level of understanding and acceptance by payers and regulators of the meaningful collaboration with patients and patient organizations. By implementing innovative approaches for patient engagement and addressing systemic barriers, PROs that better capture the outcomes most important to patients may be developed while also satisfying regulatory requirements. The path forward demands commitment from all stakeholders to recognize patients not merely as research subjects but as essential partners in advancing dermatological care.

## Data Availability

Data available upon request.
